# A Randomized, Multicenter Study Evaluating the Efficacy, Safety and Tolerability of Dapagliflozin + Gliclazide Fixed Dose Combination Over Dapagliflozin Monotherapy in Type 2 Diabetes Mellitus Patients Inadequately Controlled on Metformin Monotherapy

**DOI:** 10.7759/cureus.103676

**Published:** 2026-02-15

**Authors:** Suhas G Erande, Amar Raykantiwar, Vivek Shejole, Rashmi GR, Animesh Maiti, A.S. Veeramani Kartheek, Mohamed Hanifah, Richa Giri, Sharvil Gadve, Arindam Ray, Kunal Thakkar, Balram Sharma, Saket Kant, Abhyudaya Verma, Yogesh Yadav, Hiren Prajapati, Honey Desai

**Affiliations:** 1 Department of Diabetes and Endocrinology, Akshay Hospital & Diabetic Speciality Centre, Pune, IND; 2 Department of Medicine and Diabetology, Atharva Diabetes and Thyroid Centre, Pune, IND; 3 Department of Medicine and Diabetology, Aspiria Healthcare, Pune, IND; 4 Department of Diabetology, Lifespan Diabetes Clinic, Bangalore, IND; 5 Department of Endocrinology, Calcutta Medical College, Kolkata, IND; 6 Department of General Medicine, Andhra Medical College, Vishakhapatnam, IND; 7 Department of General Medicine, Mahatma Gandhi Medical College and Research Institute, Puducherry, IND; 8 Department of Medicine, Ganesh Shankar Vidyarthi Memorial Medical College, Kanpur, IND; 9 Department of Endocrinology, Excel Endocrine Centre, Kolhapur, IND; 10 Department of General Medicine, College of Medicine & Sagore Dutta Hospital, Kolkata, IND; 11 Department of Endocrinology, Sterling Ramkrishna Speciality Hospital, Gandhidham, IND; 12 Department of Endocrinology, Sawai Man Singh Medical College, Jaipur, IND; 13 Department of Endocrinology, Max Healthcare, New Delhi, IND; 14 Department of Endocrinology and Diabetology, Superspeciality Endocrinology and Women Care Centre, Indore, IND; 15 Department of Endocrinology and Diabetes, Dwarka Clinics, Dehradun, IND; 16 Department of Medical Affairs, Eris Lifesciences Limited, Ahmedabad, IND

**Keywords:** dapagliflozin, fixed dose combination, gliclazide sr, glycemic control (hba1c), type 2 diabetes mellitus (type 2 dm)

## Abstract

Background

Sodium glucose transporter 2 inhibitors (SGLT2i) and sulfonylurea (SU) offer an additive mechanism of action, potentially enhancing glycemic control in patients inadequately managed with metformin monotherapy. This study compared the efficacy and safety of a fixed-dose combination (FDC) of dapagliflozin and gliclazide sustained release (SR) in patients with type 2 diabetes mellitus (T2DM) poorly controlled with metformin monotherapy.

Methods

This multicentric, prospective, randomized, open-label, parallel-group, phase III clinical study was carried out at several sites around India. A total of 244 T2DM patients were randomized in a 1:1 ratio to receive either dapagliflozin 10 mg + gliclazide 60 mg SR (test arm) or dapagliflozin 10 mg (reference arm) for 16 weeks. The primary endpoint was the adjusted mean change from baseline in glycated hemoglobin A1c (HbA1c) at the end of week 16.

Results

In both arms, the baseline mean HbA1c was 8%. From baseline to week 16, the adjusted mean reduction in HbA1c was statistically significant in the FDC combination of dapagliflozin and gliclazide SR compared to dapagliflozin, with a change in mean HbA1c levels from baseline of 8.76% to 6.97% (SD: 1.02%; p-value < 0.001) and 8.80% to 7.32% (SD: 1.24%; p-value < 0.001), respectively. The FDC was well-tolerated with a favorable safety profile.

Conclusion

The FDC of dapagliflozin 10 mg and gliclazide 60 mg SR proved to be efficacious and exhibited a favorable safety profile in patients with T2DM poorly controlled with metformin compared to dapagliflozin 10 mg monotherapy.

## Introduction

Diabetes mellitus is a group of metabolic disorders characterized by chronic blood glucose elevations (hyperglycemia) caused by insulin secretion deficiency (type one diabetes [T1DM]) or insulin action defects, such as insulin resistance affecting the liver and peripheral tissues [1]. According to the International Diabetes Federation 2025 (IDF Atlas), there are 589 million adults with diabetes worldwide, with projections showing the number to rise to 853 million by 2050, and over 250 million people are unaware they have the condition; 81% of these individuals live in low- and middle-income countries [2]. A more recent report from the Non-Communicable Diseases Risk Factor Collaboration that used fasting plasma glucose (FPG) and/or HbA1c >6.5% criteria to diagnose diabetes, this survey reported the number of people with diabetes in India to be 212 million [3].

Only 23.4% of patients achieve the desired HbA1c level of less than 7.0% despite effective anti-diabetic medication [4]. Due to its glucose-lowering efficacy, low risk of hypoglycemia, weight neutrality, and cost, metformin is the preferred treatment for hyperglycemia in most diabetics. Metformin monotherapy is often inadequate for maintaining glucose levels within the target range [5]. Particularly, for long-term durable glycemic control, early combination therapy should be considered for all patients with HbA1c levels of 7.5% or higher [6]. Evidence indicates that initial combination therapy has several benefits over monotherapy, including improved β-cell function and fewer treatment failures [7]. FDC reduces pill burden and complexity of regimen, which further offers better patient compliance [8].

A dual drug therapy regimen containing dapagliflozin and gliclazide provides the additive actions, keeping hyperglycemia under control. Dapagliflozin acts by blocking the SGLT2 protein in the kidneys, consequently diminishing glucose reabsorption and augmenting glucose excretion through urine. Gliclazide is an insulin secretagogue that acts on β-cells of the pancreas and releases insulin [9]. T2DM is linked to a higher risk of cardiovascular disease, and patients with T2DM who are poorly controlled by their current antidiabetic regimen may benefit significantly from the weight loss and blood pressure decrease that is offered with dapagliflozin medication [9]. Also, a study reported that dapagliflozin, if co-administered with sulfonylureas, is pharmacokinetically compatible when observed for Cmax, AUC, Tmax, or t1/2 [10]. Therefore, FDC of dapagliflozin and gliclazide SR helps in improving the HbA1c, glycemic variability, fasting plasma glucose, and quality of life. The Indian guideline recommends starting combination therapy with SGLT2i and SUs after metformin treatment failure [11]. However, dapagliflozin with gliclazide as an FDC therapy has not been evaluated in Indian diabetic patients before. Thus, this study has been designed to evaluate the efficacy, safety, and tolerability of dapagliflozin with gliclazide FDC in T2DM patients uncontrolled on metformin monotherapy.

## Materials and methods

Study design and ethics

This phase III clinical study was multicentric, prospective, randomized, open-label, parallel-group, and comparative. The Declaration of Helsinki (2013) and the ICMR (Indian Council of Medical Research) Ethical Guidelines for Biomedical Research on Human Patients (2017) were followed during the conduct of the study. The clinical trial was prospectively registered (Reg. No.: CTRI/2023/01/049286 dated December 19, 2022) with the Clinical Trial Registry of India.

Study population

The study was carried out in ten locations across India between February and August of 2023. After an eight-week screening process, the individuals were enrolled based on predetermined inclusion and exclusion criteria, with baseline characteristics listed in Table 1. Informed consent was obtained from the patients before enrolling in the study. Eligible patients were adults aged between 18 and 65 years, non-pregnant women (women of childbearing potential who have a negative urine pregnancy test and who adhere to using an appropriate method of contraception to prevent conception during the study), with a body mass index (BMI) of <35 kg/m² and HbA1c between 8.0% and 10.0%, and patients with T2DM who are inadequately controlled on metformin monotherapy ≥1000 mg per day for at least three months.

Key exclusion criteria included eGFR <60 ml/min/1.73 m², T1DM, FPG ≥270 mg/dl, current use of thiazolidinediones, SUs, glucagon-like peptide-1 receptor agonist (GLP-1RA), dipeptidyl peptidase 4 inhibitors (DPP4i), SGLT2i, severe uncontrolled hypertension (SBP ≥150 or DBP ≥100 mmHg), recent cardiovascular events, severe liver dysfunction, active cancer, having undergone a surgical procedure within four weeks prior to signing the informed consent form, and allergy to study agents.

Study treatment

**Figure 1:**
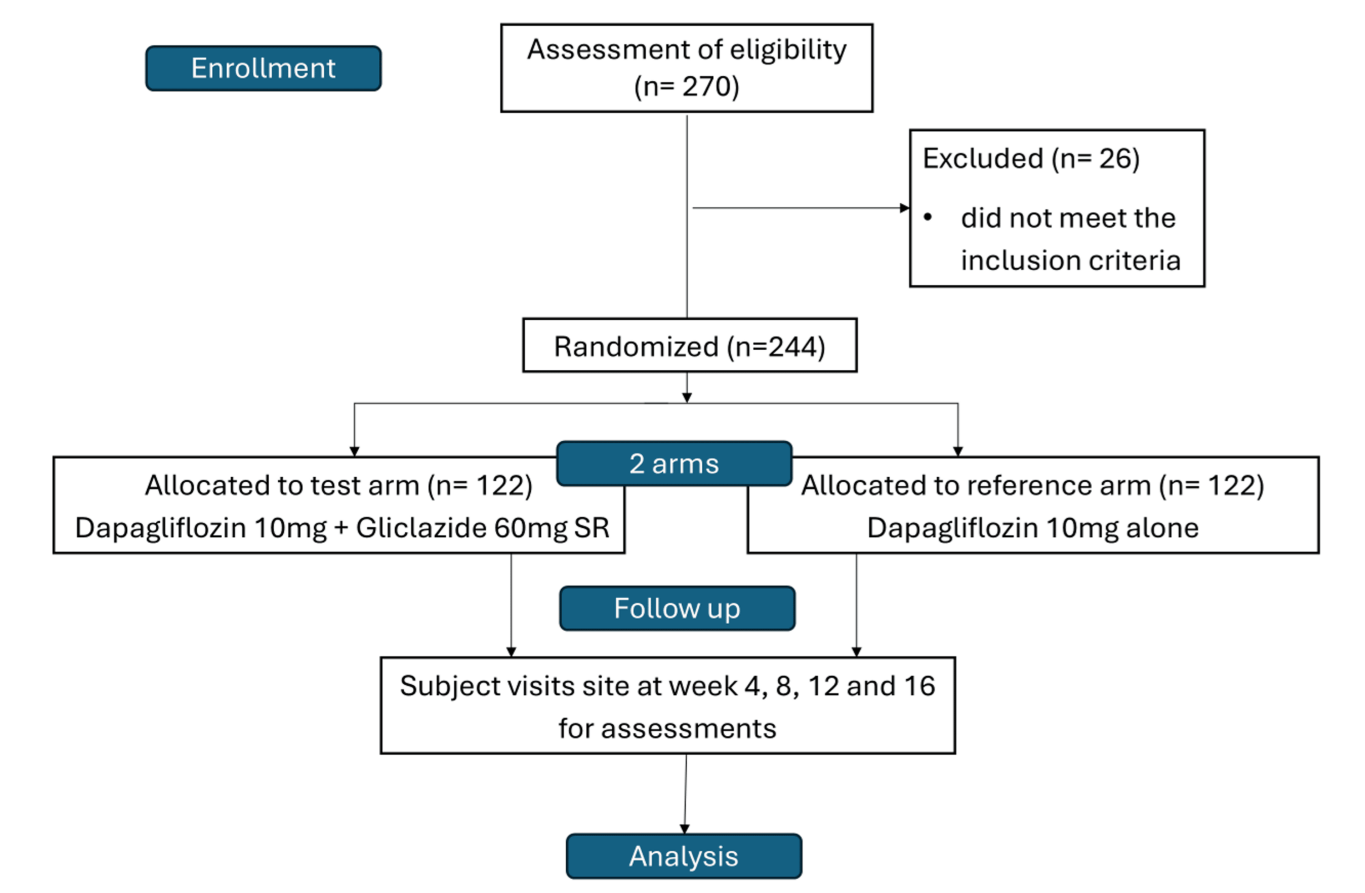
Study flow chart

Eligible patients were allocated to one of the groups in a 1:1 ratio using a simple block randomization, and a randomization code was assigned to the patients by the Interactive Web Response system. Patients in the test arm received FDC of dapagliflozin 10 mg and gliclazide 60 mg SR tablets, while those in the reference arm received dapagliflozin 10 mg, given orally every day for 16 weeks of the treatment period. All doses prescribed and dispensed to the patient during the study were recorded. The study flowchart is illustrated in Figure 1.

Study procedure

After the screening period of seven days, patients were randomly assigned to one of the two study groups in a 1:1 ratio of FDC dapagliflozin 10 mg and gliclazide 60 mg SR or dapagliflozin 10 mg to receive the treatment for 16 weeks. Following randomization, patients were monitored at 4, 8, and 12 weeks to assess FPG and 2-hour postprandial plasma glucose (PPG) levels and evaluate for any adverse effects. On completion of the treatment period of 16 weeks (112 days), patients' glycemic parameters were assessed. Furthermore, vital signs and key laboratory findings were evaluated. BMI and waist circumference were measured, and a physical examination was conducted.

Study assessment

The primary endpoint was to evaluate the adjusted mean change from baseline in HbA1c at Week 16. Secondary endpoints included mean change from baseline in FPG, 2hr PPG, and proportion of patients achieving HbA1c < 8% from baseline to week 16. The incidence of adverse events, vital signs, and laboratory test results, such as complete blood count, urine analysis, liver function test, and renal function test, was used to assess safety.

Statistical analysis

Continuous parameters were summarized as mean, standard deviation, median, minimum, and maximum, while categorical parameters were expressed as frequencies and percentages. Normality was assessed using the Shapiro-Wilk test. Within-arm changes were analyzed using a paired t-test, and between-arm differences using an independent t-test. Primary and secondary endpoints at day 112 were compared between arms using analysis of covariance (ANCOVA) with baseline values as covariates. Categorical parameters were compared using Pearson's chi-square test. All analyses were performed and validated using R programming. Statistical significance was set at p<0.05.

## Results

Among 270 patients screened across all sites, a total of 244 patients who met the eligibility criteria were randomized in a 1:1 ratio with FDC of dapagliflozin 10 mg and gliclazide 60 mg SR (n=122) and dapagliflozin 10 mg (n=122) in accordance with the randomization schedule. All patients finished the 16-week treatment period and were considered for the efficacy analysis. The safety analysis encompassed patients who had received a minimum of one dose of the prescribed treatment.

Patients in the test arm had a mean age of 48.9 years (SD: 9.1), while those in the reference arm had 49.3 (SD: 9.21) (Table 1). Demographic factors, including mean age, gender, and BMI, were not statistically significant between the arms.

**Table 1:** Baseline patient characteristics (n=244) Values were presented as mean ± SD. Height, weight, body mass index (BMI), HbA1c, fasting plasma glucose (FPG), postprandial glucose levels (PPG) at screening visit were considered. Test: FDC of Dapagliflozin 10 mg + Gliclazide SR 60 mg tablets. Reference: Dapagliflozin 10 mg tablets. N = Total number of subjects in each group. Between-group comparisons of baseline continuous variables were determined using an unpaired t-test. Categorical variables were compared using the Pearson chi-square test.

Variable	Test group (n=122)	Reference group (n=122)	Overall (n=244)	p-value
Age (years)	48.90 ± 9.10	49.30 ± 9.20	49.10 ± 9.15	0.75
Height (cm)	162.91 ± 8.57	162.72 ± 8.11	162.81 ± 8.34	0.86
Weight (kg)	68.39 ± 10.21	68.46 ± 10.67	68.42 ± 10.44	0.95
BMI (kg/m^2^)	25.69 ± 2.63	25.79 ± 3.13	25.74 ± 2.89	0.79
Waist circumference (cm)	79.19 ± 25.83	77.64 ± 26.76	78.42 ± 26.30	0.64
HbA1c (%)	8.76 ± 0.311	8.80 ± 1.02	8.78 ± 0.66	0.28
FPG (mg/dl)	172.10 ± 40.62	172.35 ± 44.09	172.22 ± 42.35	0.96
PPG (mg/dl)	251.46 ± 61.83	254.51 ± 58.73	252.98 ± 60.28	0.69

Efficacy

**Figure 2:**
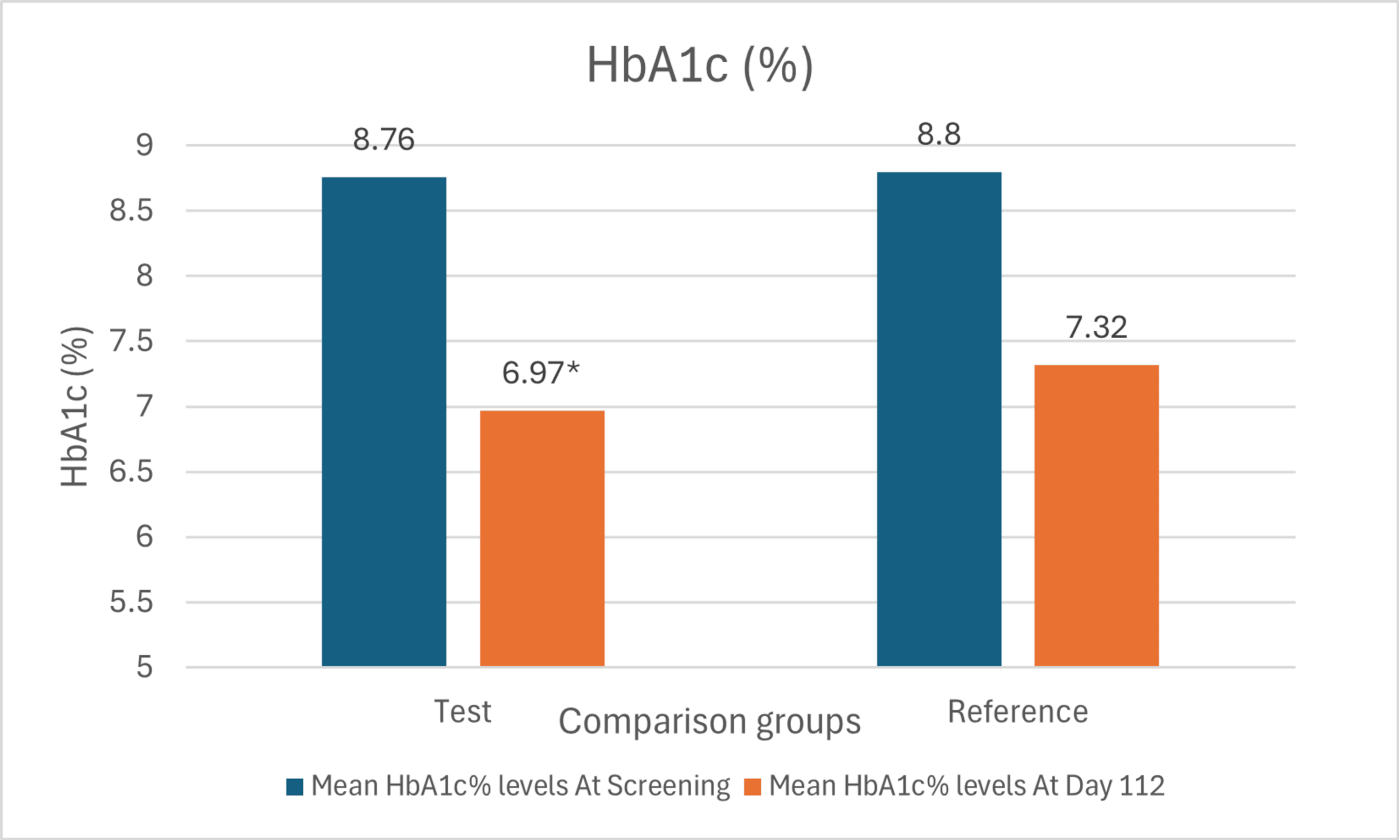
Bar chart showing mean levels of HbA1c at screening and day 112 in both arms *p-value 0.018 vs dapagliflozin 10mg monotherapy. The mean difference and 95% confidence interval (CI) denote within-group changes from baseline to Week 16, evaluated using a paired t-test. The mean difference and 95% confidence interval denote between-group comparisons, evaluated using an unpaired t-test

The mean change of HbA1c in the test arm was 1.79% (SD: 1.06%), while that in the reference arm was 1.48% (SD: 1.21%) (Figure 2). The difference in the mean change of HbA1c between the two arms was statistically significant with a p-value of 0.039.

**Figure 3:**
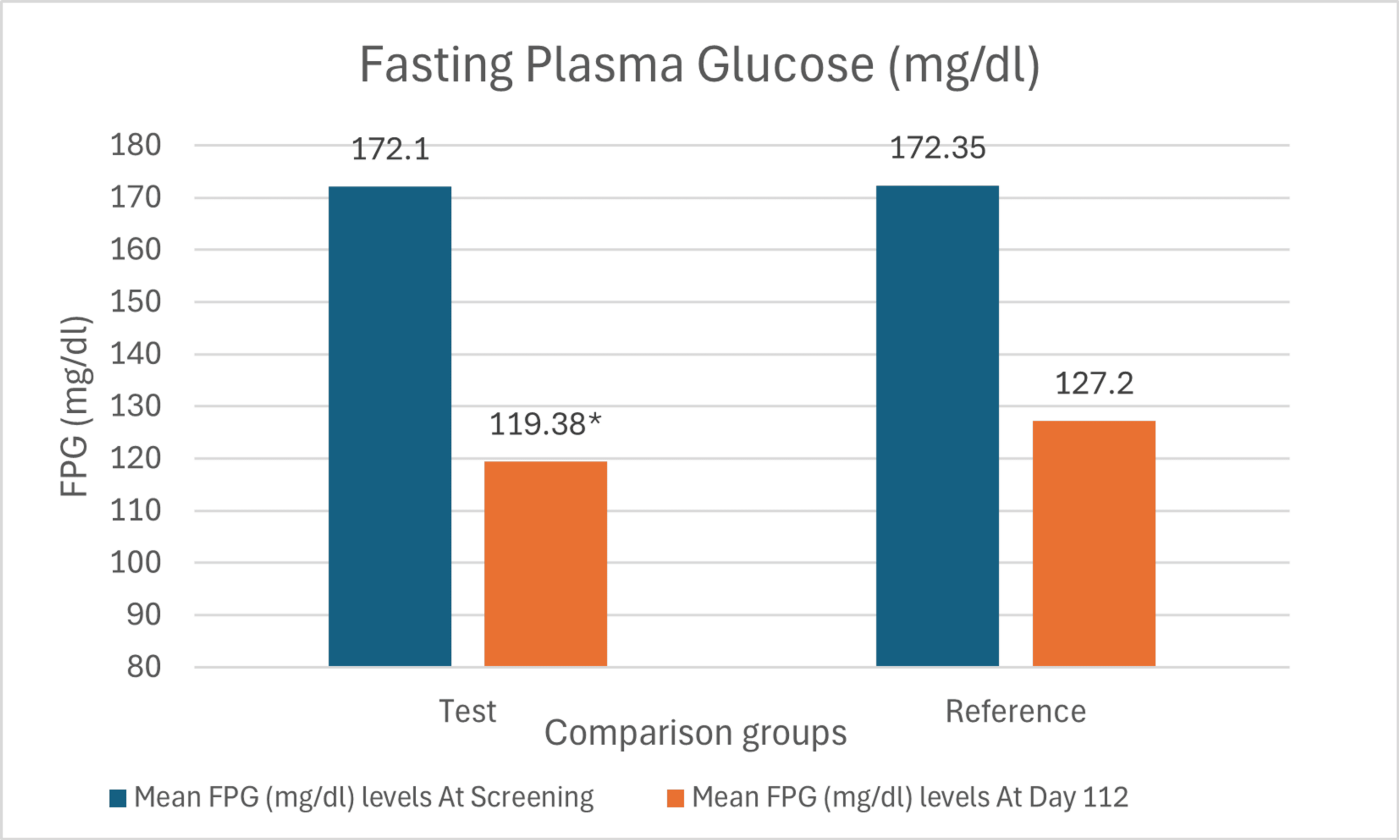
Bar chart showing mean levels of FPG at screening and day 112 in both arms *p-value 0.013 vs. dapagliflozin 10mg monotherapy. The mean difference and 95% confidence interval (CI) denote within-group changes from baseline to week 16, evaluated using a paired t-test. The mean difference and 95% confidence interval denote between-group comparisons, evaluated using an unpaired t-test.

The mean change of FPG levels from baseline in the test arm was 52.72 mg/dl (SD: 38.41 mg/dl), while that in the reference arm was 45.15 mg/dl (SD: 42.61 mg/dl) (Figure 3). The difference in the mean change of FPG levels between the two arms was statistically significant with a p-value of 0.008.

**Figure 4:**
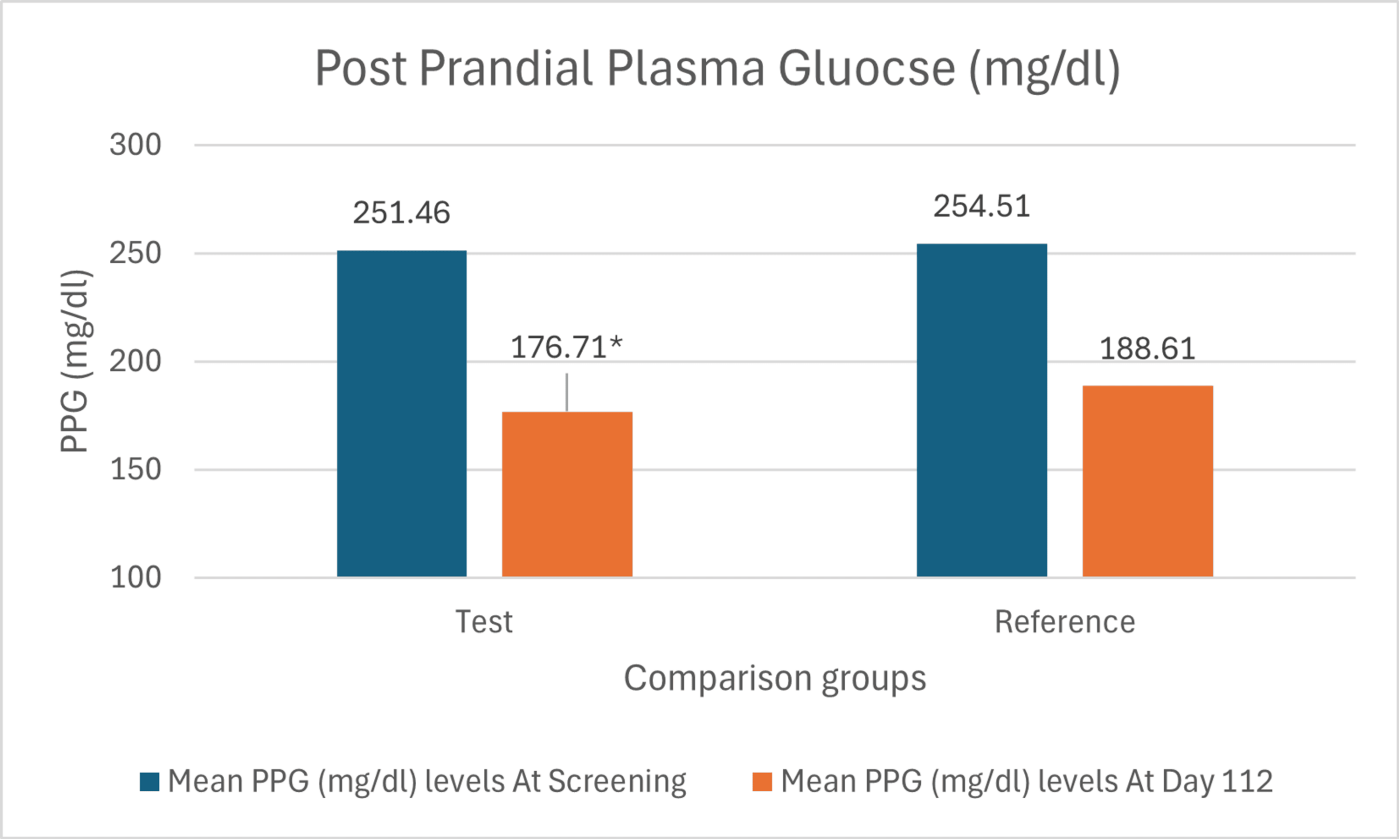
Bar chart showing mean change in PPG levels from screening to day 112 in both arms. *p value 0.044 vs. dapagliflozin 10mg monotherapy. The mean difference and 95% confidence interval (CI) denote within-group changes from baseline to Week 16, evaluated using a paired t-test. The mean difference and 95% confidence interval denote between-group comparisons, evaluated using an unpaired t-test.

The mean change of PPG levels in the test arm was 74.75 mg/dl (SD: 53.07 mg/dl), while that in the reference arm was 65.90 mg/dl (SD: 57.25 mg/dl) (Figure 4). The mean difference (-10.773 mg/dL) between test and reference arms was statistically significant with a p-value of 0.038.

In the test arm, there were 100 (81.9%) patients who achieved the criterion (HbA1c <8%), while in the reference arm, 93 (76.2%) achieved the criterion. Between-group comparisons were performed using the Pearson chi-square test. However, the variation in the proportions between the two arms was not statistically significant. For all the anthropometric parameters of patients, including height, weight, BMI, and waist circumference, the difference in mean levels between the arms at screening as well as on day 112 was statistically not significant.

Safety

There were five (4.1%) hypoglycemic events in the test arm, while there were no events in the reference arm. The difference in the proportion of events in the two arms was statistically nonsignificant. All the events were treated, and patients recovered during the observation period. There were no serious adverse events during the study.

## Discussion

The combination of gliclazide SR and SGLT2i is supported by a robust mechanistic rationale and the real-world ADD2DIA study [12]. The global ADD2DIA study, conducted across 25 centers in six countries among 537 patients, evaluated the efficacy of combining an SGLT2i with gliclazide-based treatment (± metformin) in individuals with T2DM treated for at least two years. The results demonstrated that this combination was effective (mean HbA1c reductions of −1.1% at six months) and exhibited fewer hypoglycemic events in patients with long-standing poorly controlled T2DM (mean HbA1c 8.7%) and multiple cardiovascular risk factors [13]. The current study corroborated and extended these findings, demonstrating a greater magnitude of HbA1c reduction (mean change -1.79% vs. -1.48%, p = 0.039) at week 16 compared to ADD2DIA’s -1.1% at 6 months. This superior glycemic control can be attributed to several factors: comparable baseline HbA1c values (mean 8.76-8.80% vs. ADD2DIA's 8.7%), the specific pharmacological properties of the gliclazide SR formulation, which provides sustained glucose-dependent insulin secretion when combined with SGLT2i, and the FDC formulation, which may have enhanced adherence.

The greater HbA1c reduction observed in the current study likely reflects higher baseline hyperglycemia, as greater baseline values yield larger absolute reductions [13]. The robustness of the SGLT2i + SU combination is further validated by comparison with previous studies [9,14,15]. A study by Schernthaner et al. demonstrated HbA1c reductions of 1.03% with SGLT2i (canagliflozin), metformin, and sulfonylurea over 52 weeks versus 0.66% with sitagliptin, highlighting the superior efficacy of the SGLT2i + SU combination over SGLT2i-DPP4i FDC [14].

The findings from the current study align with this superiority, as the mean HbA1c reduction of -1.79% in the FDC group substantially exceeds the sitagliptin comparator results, thereby reinforcing this therapeutic approach's robustness for glycemic control in uncontrolled T2DM patients. One of the trials by Matthaei et al. [9] showed that dapagliflozin added to metformin + SU therapy resulted in significant HbA1c reductions with a placebo-adjusted difference of -0.82% at 24 weeks and FPG improvements, which further confirmed the combination addresses HbA1c, FPG, and PPG effectively, mirroring the present study with a statistically significant difference in HbA1c (-0.31%, p=0.039 between both arms). Furthermore, significant improvements in FPG and PPG were demonstrated in FDC of dapagliflozin 10 mg + gliclazide SR 60 mg compared to dapagliflozin 10 mg alone.

The clinical relevance of the current study is particularly strengthened by the correlation with a recent phase 3 Indian study evaluating the triple FDC of dapagliflozin, glimepiride, and metformin extended-release (ER), which reported mean HbA1c reductions of 1.49% at 24 weeks in patients poorly controlled on metformin and glimepiride, further substantiating the efficacy of the SGLT2i + SUs combination in the Indian population [15]. Also, the test arm showed a numerically higher proportion of patients achieving HbA1c < 8% (81.9% vs. 76.2%). The latest ADA 2026 guidelines recommend individualized HbA1c targets, typically <7% for most adults, with targets of <6.5% for younger adults with short diabetes duration and <7.5-8% for older adults with comorbidities [1]. Considering the HbA1c target of < 7%, it might be possible that a smaller number of patients could achieve the A1c goal in this study.

The safety profile observed in the present study is noteworthy, demonstrating no new safety signals, with hypoglycemic incidence remarkably low (4.1% test vs. 0% reference). This aligns with ADD2DIA's finding of lower hypoglycemic events over two years and validates the mechanistic safety advantages of gliclazide SR, attributable to gliclazide's selective pancreatic beta-cell binding, glucose-dependent insulin secretion, and gradual sustained-release formulation [13,16]. In conclusion, the convergence of findings from diverse populations from global to Indian-specific trials provides robust, multi-layered validation of the current study results and further makes dapagliflozin and gliclazide SR FDC an evidence-based, practical, and effective second-line treatment choice in the management of T2DM, addressing both efficacy and adherence considerations that are crucial for long-term diabetes management and prevention of diabetes-related complications.

The study had several limitations. First, the 16-week treatment duration may be insufficient to evaluate long-term glycemic durability associated with this FDC. Second, the open-label design could introduce bias in subjective assessments and treatment adherence. The exclusion of patients with advanced renal impairment (eGFR <60 mL/min/1.73 m²) restricts applicability to this diabetic subpopulation.

## Conclusions

This phase III, randomized trial established that the FDC of dapagliflozin 10 mg and gliclazide SR 60 mg provides superior glycemic control compared to dapagliflozin 10 mg in reducing HbA1c levels in patients with T2DM inadequately controlled on metformin monotherapy with minimal risk of hypoglycemia. These findings highlight the superiority of the FDC of dapagliflozin 10 mg and gliclazide SR 60 mg over dapagliflozin 10 mg and present a valuable therapeutic option among Indian patients for uncontrolled T2DM management.
